# Cannabidiol oxidation product HU-331 is a potential anticancer cannabinoid-quinone: a narrative review

**DOI:** 10.1186/s42238-021-00067-z

**Published:** 2021-04-23

**Authors:** Judy Trac, J. Myles Keck, Joseph E. Deweese

**Affiliations:** 1 Department of Pharmaceutical Sciences, Lipscomb University College of Pharmacy and Health Sciences, One University Park Drive, Nashville, TN 37204-3951 USA; 2 Department of Biochemistry, Vanderbilt University School of Medicine, Nashville, TN 37232-0146 USA

**Keywords:** HU-331, Cannabinoid, Anticancer, Quinone, Cannabidiol, Topoisomerase II

## Abstract

Cannabidiol and related cannabinoids are under exploration for the treatment of a number of disease states. The cannabinoid-quinone HU-331 has been studied as a potential anticancer therapeutic. Previous studies provide evidence that HU-331 displays anticancer activity without some of the known adverse events associated with traditional anticancer agents. In this brief review, we will explore the literature related to the activity of HU-331 in purified systems, cancer cell lines, and animal models. For example, HU-331 displays inhibitory activity against human topoisomerase IIα, a known anticancer drug target. Further, in multiple cell model systems, the IC_50_ value for HU-331 was less than 10 μM. In addition, mouse model systems demonstrate the ability of HU-331 to shrink tumors without causing cardiotoxicity. In addition, we will briefly review the activity of some key analogs and derivatives of HU-331 for various disease states. Taken together, the published studies support further exploration of HU-331 for the treatment of cancer and possibly other disease states.

## Introduction

Cannabidiol (CBD) is of increasing medicinal interest as evidenced by the approval of Epidiolex by the US Food and Drug Administration. While this medication has specific indications for seizures, other applications are under exploration for CBD and other components of cannabis (Devinsky et al. 2018; Hurd et al. 2019; Mechoulam and Hanus 2002; Mechoulam et al. 2002; Nagarkatti et al. 2009; Szaflarski et al. 2018). For example, CBD has been proposed for the treatment of inflammatory diseases such as Crohn’s disease and rheumatoid arthritis (Nagarkatti et al. 2009). In some cases, clinical trials are underway to explore some of these applications, and additional potential therapeutic uses of CBD may yet to be discovered as further research continues.

**Fig. 1:**
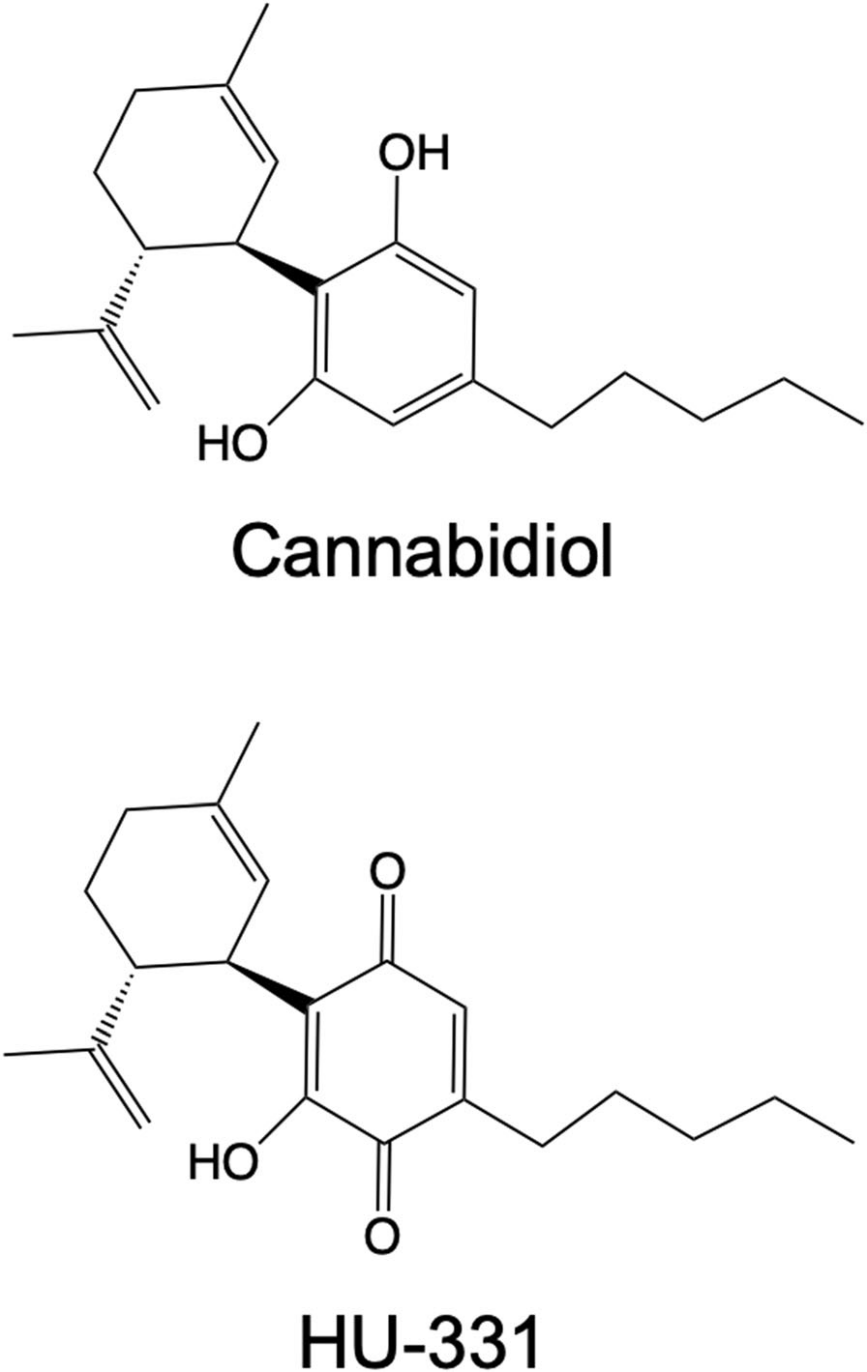
Structures of cannabidiol and HU-331

It is possible that some of the biologically relevant activity of CBD may be derived from active metabolites (Ujvary and Hanus 2016). For example, it has long been understood that CBD has many derivatives formed via oxidation and metabolism (Jiang et al. 2011; Ujvary and Hanus 2016). Among these oxidation products is the cannabidiol hydroxyquinone (CBDHQ), also known as HU-331 (Fig. 1), which has been examined as a potential anticancer therapeutic (Mechoulam et al. 1968; Kogan et al. 2004; Kogan et al. 2007a; Kogan et al. 2007b). Data from purified systems, cellular assays, and in vivo models have been published over the last 2–3 decades and indicate that HU-331 has biological activity against relevant protein targets, cancer cells, and xenograft tumors.

HU-331 has been shown to be active against the enzyme human topoisomerase II (TOP2), a known cancer drug target (Kogan et al. 2007b; Regal et al. 2014; Wilson et al. 2018). TOP2 is an essential enzyme involved in controlling DNA topology during replication, transcription, and mitosis (Nitiss 2009). Disruption of TOP2 function leads to cell death, and several anticancer agents, such as etoposide and the anthracyclines like doxorubicin, target TOP2 in order to kill cancer cells (Deweese and Osheroff 2009). The most common way TOP2 agents affect TOP2 is by stabilizing increased DNA strand breaks through a mechanism known as poisoning (Murphy et al. 2017). Etoposide and the anthracyclines are considered TOP2 poisons. This mechanism of action is associated with secondary leukemias resulting from treatment with etoposide and cardiotoxicity with anthracycline therapies (Pendleton et al. 2014; McGowan et al. 2017). However, as discussed below, HU-331 acts as a catalytic inhibitor of TOP2 and blocks enzyme activity rather than increasing DNA strand breaks (Kogan et al. 2007b; Regal et al. 2014; Wilson et al. 2018). This mechanism of action may lead to less adverse events than traditional TOP2-targeted drugs like etoposide and doxorubicin.

Further, HU-331 has also been tested in several cancer cell lines and mouse model systems (Kogan et al. 2004; Kogan et al. 2007a; Kogan et al. 2007b; Waugh et al. 2020). Evidence from these studies, which will be detailed further below, indicates that HU-331 has promising anticancer properties and may have less off-target toxicity. To that end, the intention of this review is to briefly explore the data regarding HU-331 and to consider whether this compound may be clinically viable as a therapeutic agent.

## Origin and metabolism origins of HU-331

HU-331 (or CBDHQ) is not a new compound. It was noted in the literature as early as the 1960s as being formed by oxidation of cannabidiol by potassium hydroxide resulting in the violet color that serves as the basis for the Beam test, which is used to identify the presence of cannabis (Mechoulam et al. 1968). CBD can also be oxidized by exposure to air and oxidizing agents, forming HU-331 and possibly other oxidized products (Watanabe et al. 1991; Kogan et al. 2004; Wilson et al. 2018; Waugh et al. 2020). Studies by Watanabe et al. examined the impact of HU-331 (CBDHQ) on mouse liver microsomes and demonstrated that HU-331 was isolated from a chloroform solution from CBD that stood at room temperature for 10 months (Watanabe et al. 1991). More recently, HU-331 was synthesized via Friedel-Crafts alkylation of a resorcinol derivative and subsequent oxidation using Fremy’s salt (dipotassium nitrosodisulfonate) to form the quinone (Waugh et al. 2020).

Given the reactivity of quinone compounds, Watanabe et al. also examined the ability of HU-331 to inactivate mouse CYP450 activity and found inhibition of aniline hydroxylase, p-nitroanisole o-demethylase, and aminopyrine N-demethylase activities (Watanabe et al. 1991). Across the concentration range studied (50–150 μM), HU-331 was found to inhibit these enzyme activities to a greater degree than CBD (Watanabe et al. 1991). It should be noted that CBD also inhibited these activities in a concentration-dependent manner, though to a lesser extent. In previous studies, CBD required an NADPH-generating system in order to decrease p450 activity. However, HU-331 was able to decrease p450 activity without an NADPH-generating system (Watanabe et al. 1991). They concluded that the hydroxyquinone did not require further metabolism in order to bind to CYP450 apoprotein (Watanabe et al. 1991). Further, they demonstrated that GSH and Cys were able to attenuate the effects of HU-331 on CYP450 activity in mouse microsomes, which indicates that the quinone has to be reactive and not reduced in order to have an effect (Watanabe et al. 1991).

Bornheim et al. examined CBD-mediated P450 inactivation of mouse CYP450 3A11 (Bornheim and Grillo 1998). Evidence indicates that metabolism of CBD leads to the reactive quinone metabolite (CBDHQ or HU-331), which is able to adduct directly to CYP450 3A11 as detected by mass spectrometry (Bornheim and Grillo 1998). Based upon this evidence, metabolic formation of HU-331 leads to a reactive metabolite that can covalently adduct to and inactivate CYP450 3A11.

## Biological activity and effects

### Activity against human topoisomerase II

**Fig. 2:**
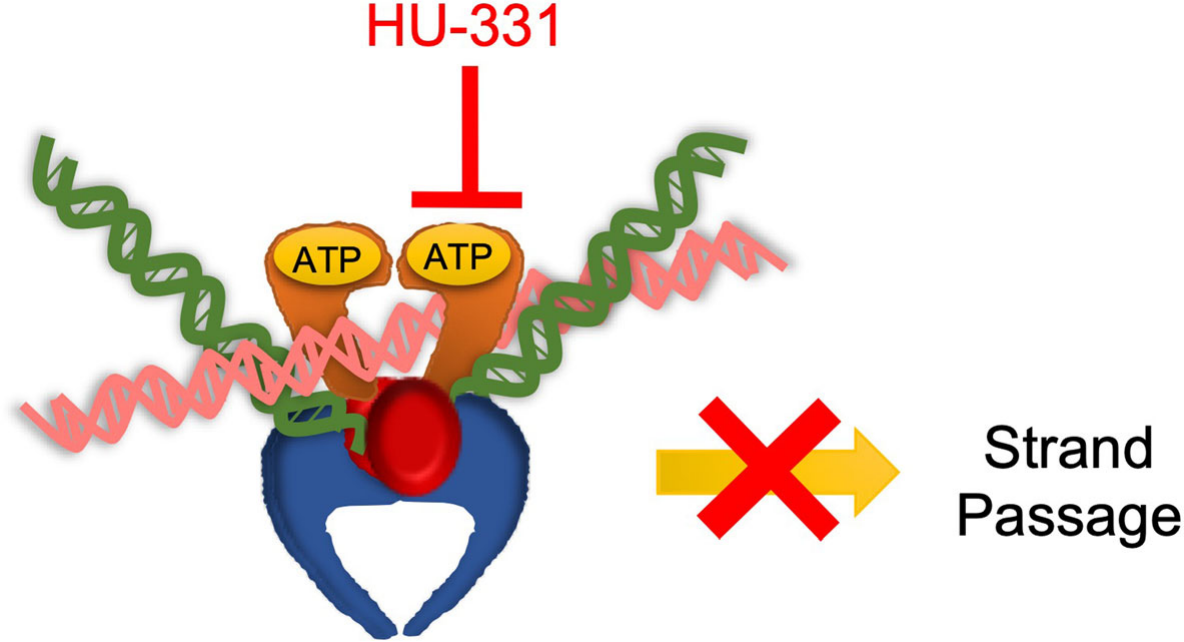
HU-331 inhibits the catalytic cycle of topoisomerase II. TOP2 (orange, red, and blue) binds to two segments of DNA (pink and green). HU-331 blocks ATP hydrolysis by the ATPase domain (orange), which results in the inability to perform strand passage and traps the enzyme on DNA

Humans encode two isoforms of topoisomerase II: topoisomerase IIα (TOP2A) and topoisomerase IIβ (TOP2B). These isoforms differ in their functional roles in cells where TOP2A is more involved in replication and mitosis, while TOP2B is more involved in transcription and chromatin regulation (Nitiss 2009). A number of widely used anticancer agents target the mechanism of topoisomerase II either by catalytic inhibition or a strand-breaking stabilizing mechanism known as poisoning (Murphy et al. 2017). These anticancer agents affect the activity of both TOP2A and TOP2B. Kogan et al. first showed that HU-331 could inhibit TOP2A-mediated plasmid DNA relaxation (Kogan et al. 2007b). Our laboratory followed upon that work and clarified that HU-331 is a catalytic inhibitor of TOP2A through inhibition of the ATPase domain (Fig. 2), which blocks the ability of the enzyme to relieve DNA supercoiling without leading to DNA strand breaks (Regal et al. 2014). This is consistent with the work of Kogan et al., who previously observed no increase in DNA strand breaks in the presence of HU-331 (Kogan et al. 2007b). A follow-up study demonstrated that this mechanism of HU-331 also works against the other human isoform of topoisomerase II, TOP2B (Wilson et al. 2018).

In addition, this latter study demonstrated that HU-331 appears to stabilize the N-terminal clamp of TOP2A and TOP2B in a closed position, which can cause the enzyme to be “locked” on the DNA (Wilson et al. 2018). Given that these isoforms share a highly similar N-terminal ATPase domain, it is reasonable to consider that the mechanism against both isoforms is the same. It is also possible that these results indicate a covalent interaction, which would be consistent with the findings of Bornheim noted above (Bornheim and Grillo 1998). However, direct evidence for adduction of HU-331 to TOP2 has not yet been demonstrated. Similar to the results from Watanabe et al., our work found that these compounds were sensitive to reducing agents and that the reduced form of HU-331 was rendered inactive against TOP2 (Regal et al. 2014; Wilson et al. 2018). Importantly, we also demonstrated that chemical oxidation of CBD results in a solution that is able to inhibit TOP2A and TOP2B in a manner similar to HU-331 (Wilson et al. 2018).

Finally, it should be noted that this compound and its analogs could affect other possible protein targets that have not yet been identified or characterized. Based upon our results, it is likely that the compound interacts with the ATPase domain of TOP2, which is a GHKL (Gyrase, Hsp90, Histidine Kinase, Mut L) ATPase domain and is shared with proteins from several diverse families such as several heat shock proteins and histidine kinases (Dutta and Inouye 2000; Jun and Kwon 2016). Additional screening assays may be needed to help identify potential protein targets.

### Activity in cellular model systems

A number of cellular systems have been used to examine the anticancer activity of HU-331 (Table 1). In 2004, Kogan et al. oxidized cannabidiol to HU-331 and then tested a series of cancer cell lines for growth inhibition using the dye-based MTT viability assay (Kogan et al. 2004). Cell lines assayed included the following: Raji (Burkitt’s lymphoma), Jurkat (T-cell lymphoma), SNB-19 (glioblastoma), MCF-7 (breast cancer), DU-145 (prostate cancer), NCI-H-226 (lung cancer), and HT-29 (colon cancer). All seven cell lines were inhibited by HU-331 in a concentration-dependent manner (Kogan et al. 2004). Specifically, Raji and Jurkat cells were inhibited by 50% at concentrations as low as 0.61–1.2 μM, while other lines ranged from ~9 to 40 μM (Kogan et al. 2004). These results support the fact that HU-331 is effective at inhibiting cancer cell growth in a wide range of cell types.

**Table 1:** Results from cell-based assays with HU-331

Cell line	Key findings/results^a^	Reference
Raji (Burkitt’s lymphoma)	IC_50_ ~0.61 μM HU-331-mediated cell death is not apoptotic	Kogan et al. (2004) Kogan et al. 2007b
Jurkat (T-cell lymphoma)	IC_50_ ~1.2 μM HU-331-mediated cell death is not apoptotic	Kogan et al. (2004) Kogan et al. (2007b)
SNB-19 (glioblastoma)	IC_50_ ~38 μM	Kogan et al. (2004)
MCF-7 (breast cancer)	IC_50_ ~9.5 μM	Kogan et al. (2004)
DU-145 (prostate cancer)	IC_50_ ~38 μM IC_50_ 9.2 μM	Kogan et al. (2004) Waugh et al. (2020)
NCI-H-226 (lung cancer)	IC_50_ <38 μM	Kogan et al. (2004)
HT-29 (colon cancer)	IC_50_ ~9.5 μM HU-331-mediated cell death is not apoptotic	Kogan et al. (2004) Kogan et al. (2007b)
BAEC (bovine aortic endothelial)	IC_50_ <1.2 μM HU-331-mediated cell death is apoptotic	Kogan et al. (2006)
HUVEC (human umbilical vein endothelial)	HU-331-mediated cell death is apoptotic Changes in gene expression observed	Kogan et al. (2006)
U-87 (human glioblastoma)	IC_50_ 9.51 μM	Macieja et al. (2019)

^a^IC_50_ values either reported directly or deduced from reported data

In 2006, Kogan et al. examined the antiangiogenic properties of HU-331 and found inhibition of blood vessel formation and proliferation of endothelial cells (Kogan et al. 2006). These results indicate that HU-331 has an effect on proliferation of vascular cells and may help decrease the ability of tumors to recruit new blood vessels. The authors examine various factors to determine how HU-331 exerts these antiangiogenic properties, including examining gene expression patterns. HU-331 did impact gene expression patterns of at least six genes, but the genes affected did not provide a clear mechanism for the antiangiogenic effects. They conclude that HU-331 induces apoptosis of vascular endothelial cells, but the exact mechanism remains to be determined (Kogan et al. 2006).

Usami and colleagues examined HU-331 in mouse liver microsomes and found that there were increases in reactive oxygen species (ROS) generation (Usami et al. 2008). In another study using mouse splenocyte cultures, researchers found that HU-331 mediates apoptosis of splenocytes via caspase-8-dependent mechanism and cellular thiol depletion (Wu and Jan 2010). According to that study, the mechanism of action does not lead to the generation of ROS as seen with doxorubicin (Wu and Jan 2010). Thus, it appears that cellular and tissue context are key to determining whether the compound contributes to ROS generation.

In 2019, Macieja et al. investigated the effect of HU-331 combined with cisplatin on U-87 human glioblastoma cells (Macieja et al. 2019). Results indicated that a combination of cisplatin and HU-331 was effective at inhibiting cancer cell growth. HU-331 has a moderate synergistic anticancer effect with cisplatin and HU-331 at low micromolar concentrations (<10 μM HU-331), which may allow for a decrease in drug doses during treatment of glioma and lower the risk of potential adverse events. They suggest that further studies are needed in order to determine if HU-331 would be beneficial in the combined treatment in glioma therapy (Macieja et al. 2019).

In 2020, Waugh et al. analyzed the effects of multiple 2-hydroxy-1,4-benzoquinone derivatives. Over 10 analogs and derivatives were tested for their antiproliferative properties and compared to HU-331 (Waugh et al. 2020). DU-145 (prostate cancer), Jurkat (human acute T cell leukemia), and Raji (human lymphoma) cells were used to study HU-331 and the analogs. Cell proliferation inhibition was measured via a XTT assay, and IC_50_ values were compared for HU-331 and the derivatives. In DU-145 cells, four compounds demonstrated higher inhibitory potency compared to HU-331. Of note, a 3-cycloalkyl derivative of 2-hydroxy-6-*n*-pentyl-1,4-benzoquinone compound called 9o drew great interest as its chemical structure has been shown to have greater chemical stability than HU-331. Focusing in on this compound, this study revealed that compound 9o had increased potency properties across all three tested cell lines when compared to HU-331 (Waugh et al. 2020). Given 9o increased solubility properties, the authors concluded that compound 9o was a candidate for further investigation as a potential anticancer agent (Waugh et al. 2020).

### Activity in animal model systems

Several sets of animal studies have been conducted as documented in Table 2. In 2004, Kogan et al. followed their cellular work with in vivo experiments. Here, nude mice were injected with HT-29 human colon cancer cells and were treated with 5mg/kg HU-331 three times a week (Kogan et al. 2004). HU-331 led to significant tumor size reduction (compared to control) using more than one route of administration (intraperitoneal, subcutaneous, and intratumoral), and this effect appeared to be dose-dependent. Together, this data revealed the ability of HU-331 to shrink HT-29 xenograft tumors across multiple doses and different administration routes (Kogan et al. 2004).

**Table 2:** Results from animal model-based experiments with HU-331

Mouse model	Cancer/tumor type	Dose and route of administration	Effect	Reference
Nude mice	HT-29 human colon cancer	5 mg/kg 3x/week, subcutaneous and intraperitoneal 5 mg/kg 3x/week, subcutaneous and intratumoral 2.5 mg/kg, intraperitoneal	Significant tumor reduction with both routes Subcutaneous has faster response than intratumoral Less effective than 5 mg/kg	Kogan et al. (2004)
Nude mice	HT-29 human colon cancer cells	15 mg/kg/week, subcutaneous	Decrease in tumor vascularization	Kogan et al. (2006)
Sabra mice		7.5 mg/kg/week	No major toxicities; mice gained weight	Kogan et al. (2007a)
Nude mice	HT-29 human colon cancer cells	5 mg/kg, 3x/week	Tumor shrinkage, weight gain, no change in ejection fraction, and no increase in cTnT	
SCID-NOD mice	Raji human B-cell lymphoma	15 mg/kg/week	Tumor shrinkage, weight gain, no increase in cTnT, nonsignificant effect on white blood cells, no evidence of cardiotoxicity	

HU-331 was also tested for inhibition of tumor angiogenesis in nude mice that were injected with HT-29 cancer cells (Kogan et al. 2006). A significant decrease in the total area occupied by vessels was seen in HU-331-treated (15 mg/kg/week, subcutaneous injection) tumors when compared to vehicle-treated tumors. Doxorubicin (2.5 mg/kg/week) was also tested and was unable to significantly affect tumor vascularization (Kogan et al. 2006).

In 2007, Kogan et al. compared the anticancer activity and adverse event profile (general toxicity) of HU-331 vs doxorubicin in vivo using additional mouse model systems (Kogan et al. 2007a). General toxicity was assayed in Sabra, nude, and SCID-NOD mice. Cardiac toxicity and myelotoxicity were also accessed. HU-331 did not change the cardiac ejection fraction or animal weight, while doxorubicin did cause a reduction in ejection fraction and weight (Kogan et al. 2007a). Further, HU-331-treated nude mice with HT-29 colon cancer xenografts demonstrated a reduction in tumor area and weight compared to control. In addition, the authors assessed cardiac toxicity, general toxicity, and myelotoxicity of HU-331 in SCID-NOD mice with xenotransplanted Raji human B-cell lymphoma. Using a 15 mg/kg weekly dose of HU-331, the tumors shrank significantly compared to control, and the mice gained weight rather than losing weight, as was the case for doxorubicin-treated mice (Kogan et al. 2007a). HU-331 did not induce an increase in cardiac troponin T (cTnT) levels and did not significantly impact blood cell counts. Further, HU-331 did not appear to generate ROS or contribute to cardiotoxicity, unlike the widely used anthracycline doxorubicin (Kogan et al. 2007a).

## HU-331 analogs and derivatives

Derivatives of HU-331 have also been developed in an effort to pursue potential therapeutics based upon this compound. An analog of HU-331 (VCE-004.8) is currently being investigated as a potential therapeutic for fibrosis in the treatment of systemic sclerosis or other fibrotic diseases (del Rio et al. 2016). This agent is the focus of an active phase 2 clinical trial, which is expected to have results by the end of 2020. Furthermore, evidence from studies of VCE-004.8 concludes that it acts on PPARγ (peroxisome proliferator-activated receptor gamma) as a partial agonist but lacks adipogenic activity reducing the metabolic disruption and inflammatory processes that are associated with obesity (del Rio et al. 2016; Palomares et al. 2018). PPARγ is a nuclear receptor that regulates adipocyte differentiation. In vivo studies show that VCE-004.8 promotes decreases in weight gain, total mass of fat, volume of adipocytes, plasma triglyceride levels, and liver stenosis in mice on a high-fat diet (Palomares et al. 2018). They also report that VCE-004.8 improved sensitivity to insulin in obese mice (Palomares et al. 2018). These findings suggest that VCE-004.8 may also have potential to be used in therapy for type 2 diabetes and obesity (Palomares et al. 2018).

Another derivative of HU-331, VCE-004.3, acts as an agonist of PPARγ and cannabinoid CB2 receptors and is an antagonist at cannabinoid CB1 receptors, making it distinct from VCE-004.8 (Del Rio et al. 2018). VCE-004.3 is believed to be a candidate for the development of novel therapies against different forms of scleroderma (Del Rio et al. 2018).

While beyond the scope of this current review, it should also be noted that a series of quinone compounds derived from cannabigerol (CBG) are also being explored for their potential therapeutic applications (Granja et al. 2012; Rodriguez-Cueto et al. 2018). This includes VCE-003 and VCE-003.2, which are being tested for neuroprotective activity and possible applications in Parkinson’s and multiple sclerosis (Granja et al. 2012; Rodriguez-Cueto et al. 2018; Burgaz et al. 2021). For a detailed review of other phytocannabinoids and their potential applications, see Hanus et al. (Hanus et al. 2016).

## Observations and conclusions

Over the last decade, CBD has gained popularity for its potential use in multiple disease states including spasticity caused by multiple sclerosis and pain caused by a multitude of medical conditions (Borgelt et al. 2013). It has been known for decades that HU-331 is a metabolite of CBD, but its functional use as a potential pharmacotherapy option was unknown until the early 2000s. Several research groups have explored the ability of HU-331 to impact protein targets, cancer cell viability, and tumor growth, as discussed above.

Collectively, the results demonstrated that HU-331 disrupted topoisomerase II function, inhibited cancer cell growth, prevented angiogenesis, and decreased tumor size in xenograft models. Unlike doxorubicin, HU-331 did not cause cardiac toxicity, which is a significant dose-limiting factor for the anthracyclines. Thus, HU-331 has the potential to be an effective cancer treatment option while potentially avoiding some of the toxic effects of agents like doxorubicin.

Recent studies demonstrate that HU-331 can work alongside traditional anticancer agents like cisplatin. However, this study was in a cellular system and may or may not translate to humans. Certainly, additional studies of HU-331 alongside other approved anticancer agents are needed. Given that HU-331 is a reactive quinone, it is also possible that it may participate in side reactions and either become neutralized or cause unforeseen adverse events. The redox capabilities of the compound have implications for route of administration and whether this compound can be co-administered with other agents. For example, reducing agents should be avoided since these may cancel out the impact of the compound.

As discussed above, derivatives of HU-331 are being explored for various disease states including cancer, fibrosis, diabetes, and obesity. These compounds show promise in a diverse set of disease states, but it should be noted that the activities seen with these analogs appear to be distinct from those observed with HU-331.

Additional data on HU-331 are needed in order to assess safety and efficacy as a potential anticancer therapeutic. For example, pharmacokinetic and pharmacodynamic information is needed to assess HU-331 stability, metabolism, and toxicity in human cellular systems and animal model systems. Currently, none of the published studies that were reviewed examine long-term adverse events in animal models. Thus, the safety and toxicity profiles remain to be clarified by additional studies.

Lastly, the off-target effects of HU-331 are still unclear. While there is evidence that HU-331 can impact the function of topoisomerase II, it is also possible that these compounds could impact other proteins inside and outside of cells. Additional cellular model systems may help clarify the impact of these compounds in specific contexts and also demonstrate whether there are toxicities that have not yet been observed.

In conclusion, HU-331 and related analogs are potentially viable anticancer agents. From an oncology perspective, these compounds work in several cellular and animal model contexts to inhibit cancer cell growth and decrease tumor size. While the exact mechanisms remain to be elucidated, this promising compound could help treat cancer while avoiding some of the toxicity of classical agents. In addition to anticancer activity, some of the analogs may also be useful in treating other disease states, and additional studies of this family of compounds are warranted.

## Data Availability

No applicable.

## Abbreviations

CBD: Cannabidiol
CBDHQ: Cannabidiol hydroxyquinone
cTnT: Cardiac troponin T
PPARγ: Peroxisome proliferator-activated receptor gamma
TOP2: Topoisomerase II
TOP2A: Topoisomerase IIα
TOP2B: Topoisomerase IIβ
